# Biological Response of Treatment with Saffron Petal Extract on Cytokine-Induced Oxidative Stress and Inflammation in the Caco-2/Human Leukemia Monocytic Co-Culture Model

**DOI:** 10.3390/antiox13101257

**Published:** 2024-10-17

**Authors:** Federica De Cecco, Sara Franceschelli, Valeria Panella, Maria Anna Maggi, Silvia Bisti, Arturo Bravo Nuevo, Damiano D’Ardes, Francesco Cipollone, Lorenza Speranza

**Affiliations:** 1 Department of Medicine and Aging Sciences, University “G. d’Annunzio” Chieti-Pescara, Via dei Vestini 31, 66100 Chieti, Italy; federica.dececco@unich.it (F.D.C.); valeria.panella@unich.it (V.P.); damiano.dardes@unich.it (D.D.); francesco.cipollone@unich.it (F.C.); 2 Uda-TechLab, Research Center, University “G. d’Annunzio” Chieti-Pescara, 66100 Chieti, Italy; 3 Hortus Novus, Canistro, 67051 L’Aquila, Italy; m.maggi@hortusnovus.it; 4 National Institute of Biostructure and Biosystem (INBB), V. le Medaglie D’Oro 305, 00136 Roma, Italy; s.bisti@team.it; 5 Department of Biomedical Sciences, Philadelphia College of Osteopathic Medicine (PCOM), 4170 City Ave, Philadelphia, PA 19131, USA; arturobr@pcom.edu

**Keywords:** saffron, saffron petal extract, inflammatory bowel disease, oxidative stress, inflammation

## Abstract

The pathogenesis of Inflammatory Bowel Disease (IBD) involves complex mechanisms, including immune dysregulation, gut microbiota imbalances, oxidative stress, and defects in the gastrointestinal mucosal barrier. Current treatments for IBD often have significant limitations and adverse side effects, prompting a search for alternative therapeutic strategies. Natural products with anti-inflammatory and antioxidant properties have demonstrated potential for IBD management. There is increasing interest in exploring food industry waste as a source of bioactive molecules with healthcare applications. In this study, a co-culture system of Caco-2 cells and PMA-differentiated THP-1 macrophages was used to simulate the human intestinal microenvironment. Inflammation was induced using TNF-α and IFN-γ, followed by treatment with Saffron Petal Extract (SPE). The results demonstrated that SPE significantly attenuated oxidative stress and inflammation by downregulating the expression of pro-inflammatory mediators such as iNOS, COX-2, IL-1β, and IL-6 via modulation of the NF-κB pathway. Given that NF-κB is a key regulator of macrophage-driven inflammation, our findings support further investigation of SPE as a potential complementary therapeutic agent for IBD treatment.

## 1. Introduction

Inflammatory bowel disease (IBD) is a chronic, specific inflammatory bowel disease that, depending on the manifestations of the disease, can be divided into ulcerative colitis (UC) and Crohn’s disease (CD). UC affects the inner lining of the colon (large intestine) or rectum, and common symptoms are diarrhoea, abdominal pain, rectal bleeding, weight loss, and vomiting, often accompanied by nutritional disorders, fever, and other manifestations. CD affects the deep layers of the intestinal wall [1]. It is characterised by pain in the right lower abdominal portion, severe diarrhoea, fatigue, weight loss, occasional bleeding, and malnutrition. In recent decades, there has been a global increase in the incidence of IBD due to industrialisation, and they have the highest incidence and prevalence in Western Europe and North America. The prevalence of IBD is increasing in developing countries such as Asia, Africa, South America, and Eastern Europe [2].

Although the pathogenesis of IBD is complex, the most widely accepted hypothesis currently involves a combination of one or more factors, such as immune deregulation caused by genetic or environmental factors, persistent imbalances of the gut microbiota, oxidative stress, and defects in the gastrointestinal mucosal barrier that allow luminal factors to penetrate the mucosa. All these elements interact with each other and contribute to the development and progression of IBD, leading to disturbances in intestinal mucosal homeostasis and distinct immunological alterations [3]. However, the exact cause of IBD is still unclear.

Most traditional therapies for IBD, such as those containing 5-aminosalicylic acid, corticosteroids, immunosuppressants, and biologic agents against tumour necrosis factor, can reduce intestinal inflammation but often have significant side effects for some patients [4,5].

In recent years, innovative therapies such as stem cell transplantation, faecal microbiota transplantation, helminth-based treatments, and microbiological therapies involving probiotics, prebiotics, or synbiotics have shown promising potential for the treatment of IBD, particularly UC [6]. Specifically, the use of probiotics, prebiotics, or synbiotics is emerging as a therapeutic strategy aimed at modulating immune responses, preserving the integrity of the colon’s mucosal lining, and positively influencing the composition of the gut microbiota and its related metabolites. These therapies target one of the key aspects of IBD, which is the imbalance in the intestinal microbiome, helping not only to prevent the onset of the disease but also to reduce its severity and progression [7].

Unfortunately, the high costs and recurrence rates of these therapies greatly limit clinical applications. The current treatments available for patients with IBD are not suitable for all patients and often have severe side effects. Moreover, certain medications can worsen inflammation and intestinal damage in patients with IBD. Since IBD is a chronic and progressive condition, the cost of its treatment adds to the socioeconomic burden. There is a need to develop new drugs that are affordable, safer, and more effective in treating both UC and CD [4,5]. Natural products, due to their varied activities, could have therapeutic effects on IBD [5]. In the last decade, numerous studies have shown that several natural products, with both anti-inflammatory and antioxidant activities, exert beneficial and therapeutic effects on IBD [8]. Among these, polyphenols, flavonoids, terpenoids, alkaloids, glycosides, quinolone compounds, coumarins, and bioactive peptides are considered important sources for the development of new drugs to counteract the deleterious effects of chronic inflammation, which characterise IBD [9,10]. In particular, the use of HBQ-Complex® (a combination of berberine, quercetin, and Hericium erinaceus), together with biotin and niacin, has been shown to effectively modulate the inflammatory response in an ex vivo model of inflamed tissue obtained from patients with UC or CD, through the downregulation of TNF-α and COX-2 and the upregulation of IL-10, suggesting a potential anti-inflammatory therapeutic approach for chronic inflammatory bowel diseases [11].

Recent studies have found that many polyphenols maintain the integrity of intestinal tight junctions (TJs), although information is still limited. For this reason, new studies would be desirable [12]. Suzuki and collaborators reported that kaempferol, a naturally occurring flavonoid, showed a protective effect on gut barrier integrity through modulation of the expression of peripheral adaptor proteins such as zonula occludens (ZO) associated with the actin cytoskeleton and other signalling proteins such as Claudina and occludin [13].

Nuclear factor κB (NF-κB), a transcription factor, is the hallmark of the inflammatory response. Several studies have shown that the overexpression of proinflammatory cytokines, such as tumour necrosis factor α (TNF-α) and interleukin (IL)-1β, leads to the activation of NF-κB contributing to the etiopathogenesis of IBD through impairment of intestinal barrier function. TNFα, interferon γ (IFN-γ), and interleukins are well-known for their indisputable role in regulating the integrity of tight junctions [14]. TNF-α plays a crucial role in the internalisation of occludin, leading to increased intestinal permeability. Moreover, TNFα stimulates the NFκB signal transduction pathway, an important mechanism in regulating tight junctions. IFNγ also increases barrier permeability by reducing the expression of ZO-1 and occludin through an adenosine monophosphate-activated protein kinase (AMPK)-dependent pathway, independent of cellular energy levels. The simultaneous presence of both cytokines has a detrimental effect on intestinal integrity by causing the dissociation of tight junction proteins [15]. Fbw7, also known as Fbxw7, is a type of E3 ubiquitin ligase complex which plays a crucial role in modulating the NF-kB signalling pathway. The expression level of Fbw7 negatively correlates with NF-kB signalling activity, and deletion of Fbw7 activates the NF-kB signalling pathway. Li H et al. have shown that genetic deletion of Fbw7 in the mouse intestinal epithelium worsens DSS-induced colitis, indicating that FBW7 plays a protective role in acute colitis by modulating the NF-kB pathway. Fbw7 suppresses the expression of IKK, inhibits IκB-α phosphorylation, and subsequently inhibits the phosphorylation of the p65 subunit, thus suppressing the NF-κB signalling pathway [16]. 

Every year, the food industry generates large amounts of by-products. Recently, there has been a growing interest in re-evaluating waste products as they are considered a suitable source of bioactive molecules for healthcare use [17]. In this regard, the waste products of the saffron plant, *Crocus sativus* L., specifically the petals, should be explored. Our previous work has reported that Saffron Petal Extract (SPE), downregulating the expression of the ubiquitin FBW7, inhibits the degradation of the IKBα subunit and keeps NF-κB in an inactive state, leading downstream to a reduction in the expression of pro-inflammatory proteins of iNOS, COX-2, and HO-1 in the LPS-stimulated Caco-2 monolayer cell line [18,19]. Several in vitro models have been created to understand better IBD origin, pathophysiology, and prospective therapy approaches. Many of them employed the Caco-2 cell line because differentiated Caco-2 cells could reflect many properties of mature enterocytes in the intestinal epithelium, such as a brush border with microvilli, tight junction development, synthesis of typical digestive enzymes, and transporters [20].

Therefore, in the present study, we used an in vitro transwell model of intestinal inflammation to evaluate the possible role of SPE on cellular mechanisms mediated by the transcriptional factor Nf-Kb, which significantly reduces the expression of critical proteins essential in regulating intestinal barrier permeability.

## 2. Materials and Methods

### 2.1. Saffron Petal Extract Protocol

**Figure 1:**
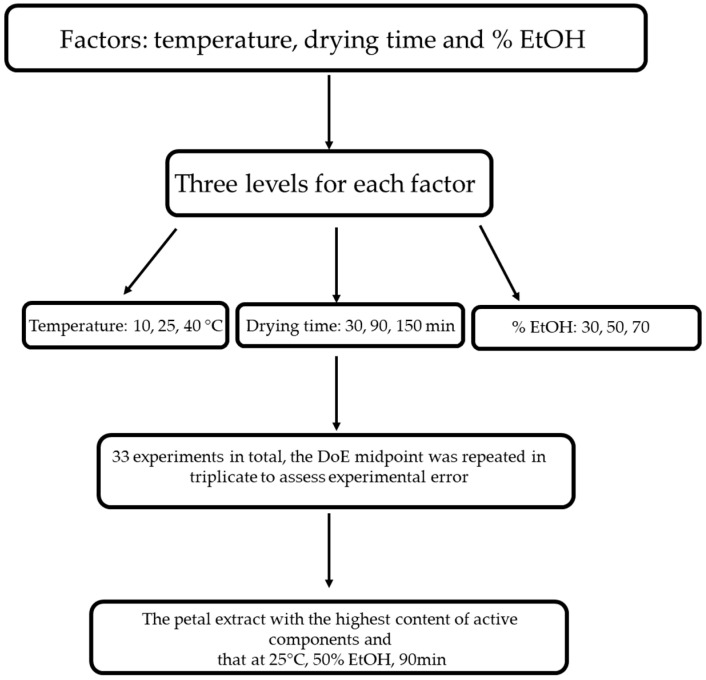
Design of experiments (DoE) flowchart of optimizing the tepal extraction process.

A reverse-phase HPLC analysis was performed for the biofunctional characterisation of saffron petals (Figure 1). The petals were dried in an oven at 50 °C for 2 h, then placed in a flask and minced. Then, 100 mg of powder was weighed and placed in contact with different extracting mixtures: H_2_O/EtOH. The mixtures considered are the following: 70 ethanol/30 water *v/v*; 50 ethanol/50 water; 30 ethanol/water. The extraction was carried out under magnetic stirring in a temperature-controlled water bath. We tried different extraction times and different bath temperatures. As regards the temperature, the experiment was conducted at 10 °C, 25 °C, and 40 °C, while the duration of the extraction was varied from 30 min to 90 min to 150 min. At the end of each experiment, the samples were placed in a centrifuge at 3000 rpm for 5 min. The supernatant was removed and filtered with a 0.20 μm nylon filter. The analysis used a reversed-phase HPLC system equipped with a photodiode array UV-Vis detector. A C18 5 μm column, Kinetec EVO, was used as the chromatographic column. The eluent phase is deionised and demineralised water acidified with 0.01% phosphoric acid and acetonitrile. The analysis is in gradient: we started from an initial condition of 95% water and 5% acetonitrile, and in 30 min, we reached 0% water and 100% acetonitrile. The chromatograms obtained were acquired at 2 different wavelengths: 265 nm for the glycosylated derivatives of campherol, the most numerous compounds, and 520 nm for the anthocyanins. By analysing the different chromatograms, it was possible to identify the best experimental conditions, i.e., those that guaranteed the maximum quantity of extracted substances. These conditions are 25 °C, 90 min, and 50 ethanol/50 water. Finally, an extract obtained with these conditions was dried to eliminate the ethanol and was used for in vitro tests.

### 2.2. Cells Culture

The Caco-2 cell line was used, obtained from a human colorectal adenocarcinoma (ATCC, Manassas, VA, USA) cultured in DMEM (Dulbecco’s Modification of Eagle’s Medium, Corning, Manassas, VA, USA) containing 4.5 g/L glucose, sodium pyruvate and 25 mM HEPES, 10% (*v/v*) heat-inactivated foetal bovine serum (Corning, Manassas, VA, USA), 1% (*v/v*) nonessential amino acids (NEAA) (Gibco, Thermo Fisher Scientific, Waltham, MA, USA), 1% (*v/v*) penicillin/streptomycin (Corning, Manassas, VA, USA), and 1% (*v/v*) of l-glutamine (Corning, Manassan, VA, USA). The THP-1 cell line was obtained from a human Acute Monocytic Leukemia (ATCC, Manassas, VA, USA) cultured in RPMI-1640 (Sigma-Aldrich, St. Louis, MO, USA) containing sodium bicarbonate, 10% (*v/v*) heat-inactivated foetal bovine serum (Corning, Manassas, VA, USA), 1% (*v/v*) penicillin/streptomycin (Corning, Manassas, VA, USA), and 1% (*v/v*) of l-glutamine (Corning, Manassan, VA, USA). Both cells were grown in T75 cm^2^ flasks (Corning, Oneonta, NY, USA) in 5% CO_2_ at 37 C in a 95% humidified atmosphere until the experiment.

For the experiments, the co-culture model of Caco-2/THP-1 macrophage was used. The co-culture was prepared according to the modified protocol described by Schnur et al. [21]. In this co-culture system, the epithelial cells (Caco-2) are placed in the upper compartment, mimicking the environment of the human intestinal epithelium. In contrast, the immune cells (THP-1) are added to the lower compartment, representing the underlying tissue. The two cell types do not come into direct contact. Still, they communicate and interact by exchanging soluble factors, mimicking the interplay between the human body’s intestinal epithelium and immune system. In brief, Caco-2 cells were seeded at Trans-well insert plates (9.6 0.4 μm pore size Falcon, Corning, Manassas, VA, USA) in a concentration of 3.75 × 10^5^ cells/cm^2^ and cultured for 21 days until cells were fully differentiated. The culture medium was changed every 2–3 days. THP-1 monocytic cells were seeded onto 6-well plates (2 × 10^6^ cells/well) and differentiated in macrophages using 50 ng/mL PMA (2-Mercaptoethanol, phorbol 12-myristate 12-acetate) for 3 days. The cells were then further incubated in a fresh medium without PMA for two days. After replacing the media with complete DMEM, inserts with Caco-2 were added into plates containing THP-1. TNF-α and INF-γ at 50 ng/mL were added to the basolateral side, and after 3 h of incubation, SPE (50 µg/mL) was applied to the apical side of the insert. SPE was also added to the insert without previous stimulation with cytokines. After 24 h of incubation, cells were collected for analysis.

### 2.3. Cell Viability Assay

The possible cytotoxic effects of SPE (range 50 ng/mL–1 mg/mL) were examined for undifferentiated (2-day culture), differentiated (15-day culture to form monolayers) Caco-2 cells, and THP-1 cells at 24 and 48 h using an MTT assay. Cells were seeded on 96-well plates at a density of 8 × 10^3^ cells/well, cultured, and treated according to the method described above [22]. A total of 20 µL of MTT was added at a concentration of 0.5 mg/mL after medium (200 µL) was added to each well. The plates were incubated at 37 °C for 4 h to dissolve the formazan that had formed. The solution (220 µL) was removed from each well and 150 µL of DMSO was added. Reduced MTT was measured on an ELISA reader (Bio-Rad, Hercules, CA, USA) at a wavelength of 570 nm. Values are expressed as a percentage of the control value.

### 2.4. Alkaline Phosphatase Activity and Protein Content

The Alkaline Phosphatase Assay Kit (MAK447, Sigma-Aldrich, St. Louis, MO, USA) measuring alkaline phosphatase (ALP) activity was used to assess cell differentiation into enterocytic cells according to the manufacturer’s protocol. Cell culture media of Caco-2 was mixed with a working reagent; subsequently, absorbance was measured at 405 nm immediately (T = 0 min) and again after 4 min (T = 4 min) with the microplate absorbance reader at 405 nm using a GO microplate spectrophotometer (Thermo Fisher Scientific, Waltham, MA, USA). ALP activity was normalised for protein content using the Pierce BCA Protein Assay Kit (Thermo Fisher Scientific).

### 2.5. Transepithelial Electrical Resistance Measurement

The integrity of the cell monolayer in the Caco-2/THP-1 co-culture on an insert was assessed using transepithelial electrical resistance (TEER) measurements. These measurements were carried out with an epithelial voltohmmeter (EVOM2; WPI, Berlin, Germany) in triplicate, immediately following medium replacement, in accordance with the manufacturer’s instructions. The final TEER value (TEER_final) was calculated by subtracting the resistance of the blank (R_blank)—representing the resistance of the semipermeable membrane without cells—from the resistance measured across the sample (R_sample). This value was then multiplied by the effective growth area (A) of the insert.

TEER final [Ω × cm^2^] = (R sample − R blank) [Ω] × A [cm^2^]

### 2.6. ROS Detection

The NBT (nitroblue tetrazolium) assay was conducted as previously described to measure intracellular ROS levels [22]. Briefly, after every treatment, cells were incubated with 0.1 mg/mL NBT in prefiltered culture medium for three h at 37 °C; then, they were rinsed three times with methanol. The quantity of NBT-formazan produced is an indicator of intracellular superoxide anion generation and can be measured spectrophotometrically (SpectraMax^®^ 190, Molecular Devices, San Jose, CA, USA) at 630 nm after solubilisation of the crystals in 200 µL of 2 M KOH/DMSO solution. The results were shown through an NBT reduction (SI) stimulation index, calculated as the optical density (OD) ratio of control and treated cells. The SI index for the control was considered to be equal to one.

### 2.7. Nitric Oxide Synthase (NOS) Activity

The oxyhaemoglobin assay was conducted to detect nitric oxide production from NOS, as detailed in the previous report [23]. The reaction mixture for assessing NOS activity consisted of CaCl2 (1.6 mM), l-arginine (10 µM), calmodulin (11.6 mg/mL), tetrahydrobiopterin (6.5 µM), dihydronicotinamide-adenine dinucleotide phosphate (NADPH, 100 µM), and oxyhaemoglobin (3 mM) in 4-(2-hydroxyethyl)-1-piperazineethanesulfonic acid (HEPES, 100 mM) at pH 7.5, in a final volume of 1 mL. iNOS activity was evaluated under calcium-free conditions. The detection of methaemoglobin, the product of the reaction between nitric oxide and oxyhaemoglobin, was performed at 576 nm (*e* = 12.000 M^−1^·cm^−1^).

### 2.8. RNA Extraction, Reverse Transcription, and Real-Time PCR

Cells were collected in 1 mL of QIAzol lysis reagent (Qiagen, Hilden, Germany), and total RNA was isolated according to the manufacturer’s protocol. The concentration of total RNA was assessed with a NanoDrop 2000 UV-Vis spectrophotometer (Thermo Fisher Scientific, Waltham, MA, USA), and one µg of total RNA was transcribed into cDNA using a QuantiTec Revers Transcription Kit with integrated removal of genomic DNA contamination (Qiagen, Hilden, Germany), following the manufacturer’s instructions. The cDNA was used for real-time PCR assays, run in triplicate using GoTaq qPCR Master Mix (Promega, Madison, WI, USA), as reported previously [22]. The following conditions were used: 2 min incubation at 95 °C; 40 cycles consisting of 30 s at 95 °C; 60 °C for 1 min; and 30 s at 68 °C. Human-specific primer pairs were used to evaluate the expression of target molecules (Table 1).

Relative expression of each gene was normalised by the 18s gene using the ΔCt method, where ΔCt = Ct_(COX-2,iNOS,HO-1,FBW7,IL-1*β*,IL-6)_ − Ct_18s_. Relative fold changes in gene expression were determined by the 2^−ΔΔCt^ method, where ΔΔCt = ΔCt_experimental sample_ − ΔCt_control sample_.

### 2.9. Western Blot Analysis

Western blot analysis was carried out as described previously [24], using the following antibodies: IpKB alpha (NFKBIA) (OTI1D4; 1:400), FBXW7 (OTI6B1; 1:1000), COX-2 (D5H5; 1:800; Cell Signalling, Danvers, MA, USA ), and β-actin (sc-47778, 1:400; Santa Cruz Biotechnology, Inc., Dallas, TX, USA), occludin (sc-133256; 1:500; Santa Cruz Biotechnology, Inc., Dallas, TX, USA), ZO-1 (A8449; 1:600; Antibodies.com, Cambridge, UK), P65 (D14E12; 1:800; Cell Signalling, Danvers, MA, USA), pP65 (93H1; 1:600, Cell Signalling, Danvers, MA, USA), HO-1 (sc-390991; 1:250; Santa Cruz Biotechnology). The membrane was then incubated for two h at room temperature with goat anti-mouse secondary antibody (Sc-2005; 1:2000; Santa Cruz Biotechnology) or polyclonal goat anti-rabbit secondary antibody (Sc-66931; 1:5000; Santa Cruz Biotechnology). The nitrocellulose has been scanned via a computerised densitometric system (Bio-Rad Gel Doc 1000, Milan, Italy). Protein concentrations were normalised to the housekeeping proteins β-actin to adjust for variability in protein loading and expressed as a percentage of vehicle control.

### 2.10. Cytokines Level Measurement

The levels of Serum IL-1β and IL-6 were analysed using enzyme-linked immunosorbent assay (ELISA) kits from Proteintech (Proteintech Group, Inc., Rosemont, IL, USA; IL-1β—cat. no.KE00021) and Proteintech (Proteintech Group, Inc., Rosemont, IL, USA; IL-6—cat. no.KE00139) following the manufacturer’s protocols. Samples were tested in triplicate, and the results were standardised by comparing them with a standard curve.

### 2.11. Measurement of PGE2 Release

Following the manufacturer’s instructions, the cell culture medium was collected at specified intervals for measuring PGE2 using an enzyme immunoassay (Arbor Assays, Ann Arbor, MI, USA). Briefly, control and samples were added to each well and incubated for 15 min at room temperature and overnight at 4 °C with primary antibody and conjugate. After washing, substrate solution was added to each well for 30 min at room temperature. Finally, ‘stop solution’ was added, and the optical density of each well was determined within 30 min using a microplate reader (wavelength 450 nm). The standards used were 12.5–400 pg/mL (detection limit of 16.8 pg/mL) for PGE2 (sensitivity of 10.9 pg/mL).

### 2.12. Immunofluorescence Staining of Occludin and ZO-1

In this experiment, 114,000 cells/well were seeded on round glass coverslips placed into 24-well plates in duplicate and cultured for 21 days until cells were fully differentiated. On day 21, the cells were fixed with 4% formaldehyde for 10 min, then washed in Phosphate Buffer Saline with 0.1% of Tween 20 (PBS-T) once for 5 min. They were incubated for 10 min with 0.05% Triton X-100 for cell permeabilisation at 4 °C and blocked 30 min in 3% of BSA at room temperature. Subsequently, the cells were incubated with primary antibodies overnight at 4 °C diluted in 1% of BSA. The day after, cells were washed three times in PBS-T and incubated with related secondary antibodies for one hour at 4 °C, washed three times in PBS-T and incubated with 4′,6-diamidino-2-phenylindole (DAPI) for 15 min at room temperature. Finally, the round glass coverslips were glued to glass microscope slides. The primary antibodies used were occludin (sc-133256; 1:50; Santa Cruz Biotechnology, Inc., Dallas, TX, USA) and ZO-1 (A8449; 1:50; Antibodies.com, Cambridge, UK). The secondary antibodies used were Alexa Fluor 594 goat anti-mouse IgG (A11005; 1:200; Thermo Fisher Scientific, Waltham, MA, USA) and Alexa Fluor 488 goat anti-rabbit IgG (H + L) (A11008; 1:200; Thermo Fisher Scientific, Waltham, MA, USA). All images were obtained using a Zeiss LSM800 confocal microscope (Carl Zeiss Microscopy GmbH, Munich, Germany).

### 2.13. Statistical Analysis

All results were expressed as means ± standard deviation. Statistical significance was calculated by one-way analysis of variance (ANOVA), and *p* < 0.05 values were considered statistically significant.

## 3. Results

### 3.1. Extraction SPE

**Figure 2:**
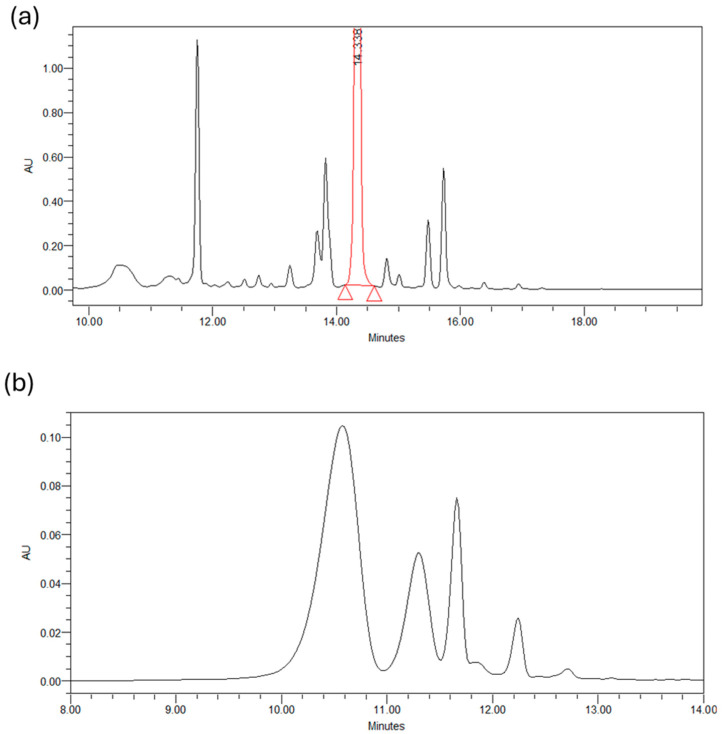
(**a**) Chromatogram of a petals extract at 265 nm. (**b**) Chromatogram of a petals extract at 530 nm.

Below are the chromatograms obtained from the chromatographic analysis of petal extracts (50/50 *v/v*, H_2_O/EtOH, 25 °C, 90 min) at two different wavelengths: 265 nm and 530 nm. Figure 2a shows the profile of glycosylated derivatives of kaempferol present in the petals, with about 20 distinct compounds identified. Kaempferol 3-O-sophoroside was the most abundant compound found in the SPE, followed by quercetin 3-O-sophoroside, isorhamnetin 3-O-glucoside, and kaempferol 3,7,40-O-triglucoside. The high content of kaempferol glycosides was based on the results reported in the literature and detected in *C. sativus* L. petals. Figure 2b shows the chromatogram at 530 nm of the four most abundant anthocyanins present in the petals: delphinidin-3,5-di O-glucoside, petuidin-3,5-di O-glucoside, delphinidin 3-O-glucoside, malvidin 3,5-di O-glucoside, and petuidin 3-O-glucoside [25].

### 3.2. Effects of SPE on Cell Survival and Oxidative Stress

**Figure 3:**
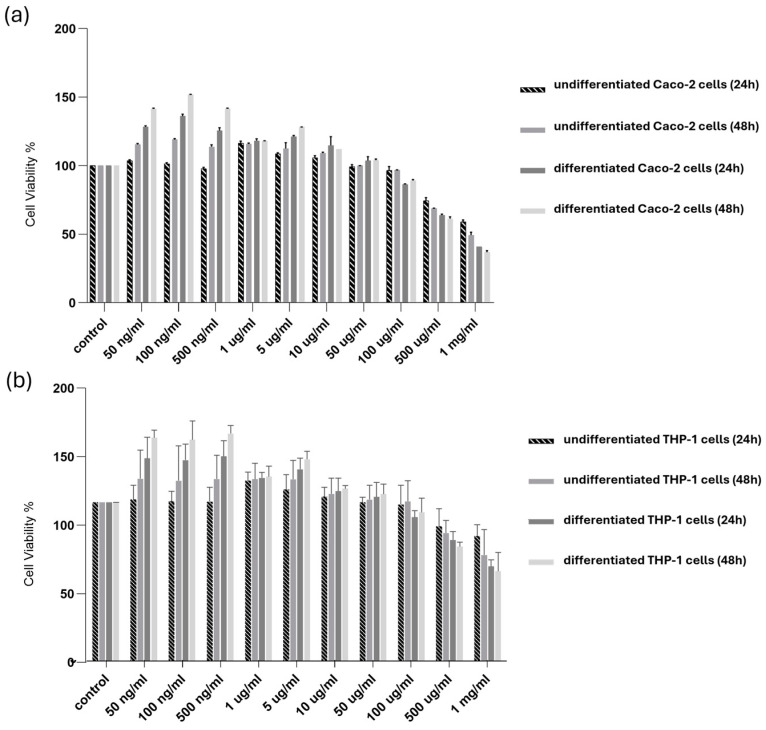
Cytotoxicity of SPE in differentiated and undifferentiated Caco-2 and THP-1 cells. (**a**) Undifferentiated and differentiated cultured (**a**) Caco-2 and (**b**) THP-1 cells were treated with SPE (range 50 ng/mL–1 mg/mL) (24 and 48 h), and cell viability was determined using an MTT assay. Cell survival (100%) was assigned to the control without treatment, and changes in cell viability relative were calculated. The data are presented as the mean ± SD (*n* = 3). SPE, saffron petal extract; MTT, 3-(4,5-dimethylthiazol-2-yl)-2,5-diphenyltetrazolium bromide.

The possible cytotoxic effects of SPE were examined for undifferentiated and differentiated Caco-2 and THP-1 cells. The cells were treated with SPE for 24 and 48 h. As reported in Figure 3, our findings revealed evident cytotoxicity in the survival of the differentiated and undifferentiated cells only for concentrations above 500 µg/mL.

**Figure 4:**
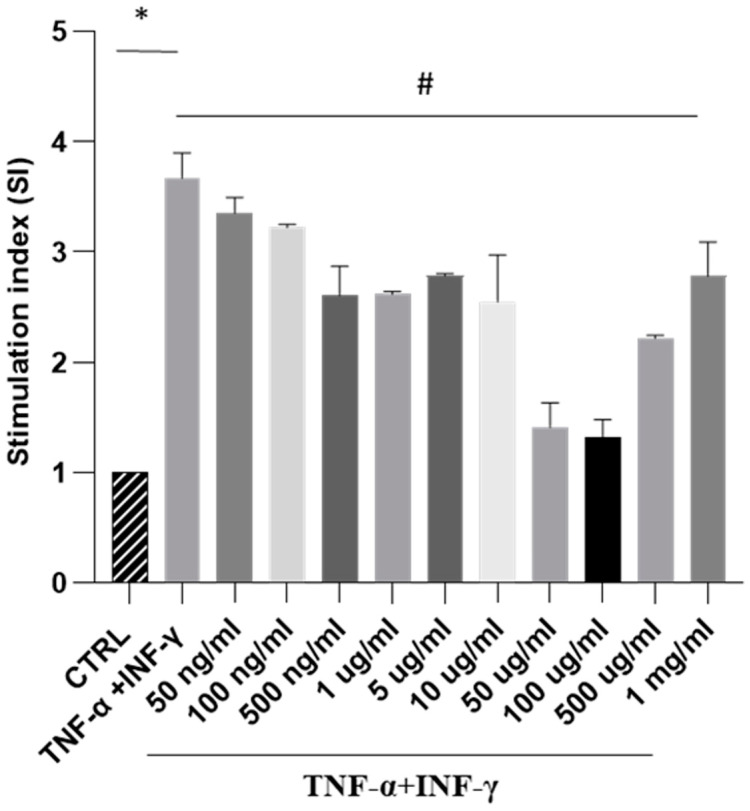
Measurement of SPE’s antioxidant activity against oxidative stress through NBT testing in a co-culture system. NBT assay visualises formazan crystal formation in Caco-2 cells treated with INF-γ and TNF-α plus SPE (50 ng/mL–1mg/mL). Results are registered as stimulation index (SI). SI value of 1 was assigned to control cells. Each bar represents ± SEM (n = 3). * *p* < 0.005 vs. CTRL; # *p* < 0.05 TNF-α + INF-γ.

Next, our focus was to analyse the impact of SPE on ROS generation in a cellular model of intestinal inflammation. In this study, Caco-2 cells triggered with THP-1 differentiated cells treated with TNF-alpha and IFN-gamma show an inflammatory response like intestinal inflammation. Our results showed that exposure to cytokines significantly increased ROS formation. In contrast, treatment with increasing concentrations of SPE decreased intracellular ROS production in a dose-dependent manner, up to a concentration of 100 µg/mL of SPE (Figure 4). These findings indicate that SPE effectively reduced ROS production in cells. Moreover, our data revealed that treating cells with SPE concentrations above 50 µg/mL significantly reduced O_2_^−^ generation by 69% compared to activated cells. Therefore, we selected concentrations of 50 µg/mL of SPE for subsequent experiments.

### 3.3. Intestinal Barrier Function

**Figure 5:**
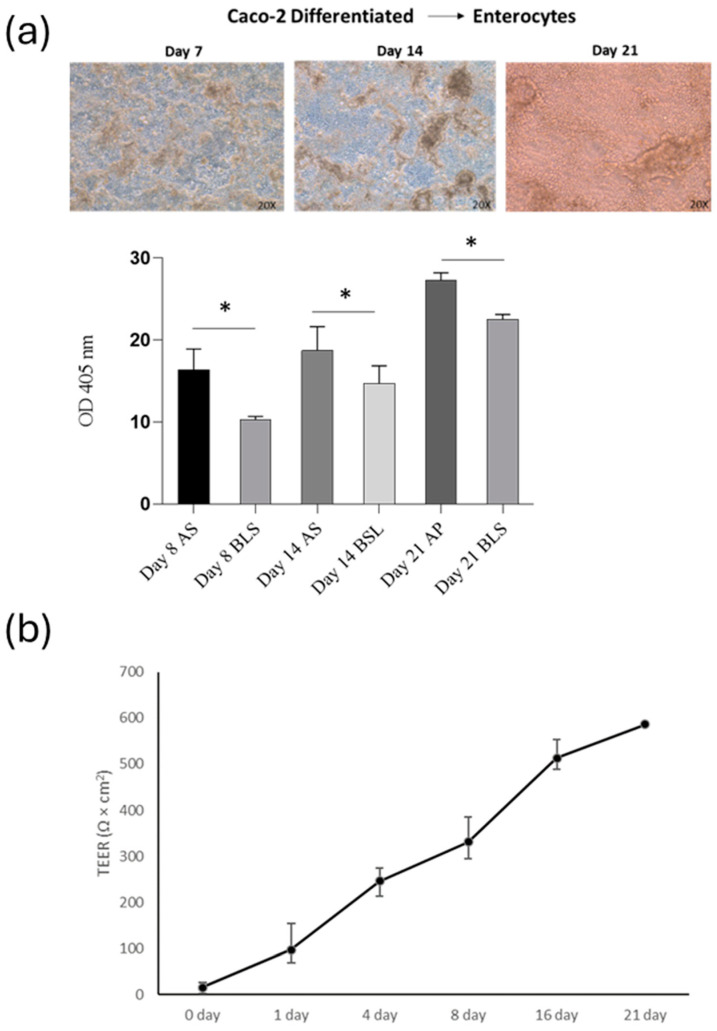
Analysis of intestinal barrier function (**a**) Alkaline phosphatase activity of apical/basolateral (AP/BL) in Caco-2 cell during 21-day incubation. Cells were analysed eight days after reaching the confluence, 14 days post-confluency (differentiated) and 21 days post-confluence. (**b**) Changes in TEER values of co-cultured Caco-2 and THP-1 cells incubated for 21 days on Transwell. Values obtained from four experiments, each performed in triplicate, are shown. * *p* < 0.05.

This study aimed to determine if SPE (at a concentration of 50 µg/mL) can reduce inflammation and prevent the breakdown of the intestinal barrier caused by cytokines. First, to assess the level of differentiation of Caco-2 cells, we measured the activity of alkaline phosphatase (ALP), a specific marker of the cells’ brush border. We found that ALP activity was low during the active growth phase of the cells. However, as the cells matured, the level of ALP activity increased significantly in 14-day-old cell cultures and plateaued in 21-day-old cultures (as shown in Figure 5a).

After assessing the impact of THP-1 cells on the Caco-2 barrier, TEER was measured at 0, 1, 4, 8, 16, and 21 days of co-culture (Figure 5b). Throughout the development of the co-culture model, the TEER value was widely used to measure the tight junction strength of the cell monolayer, as well as to assess the tightness of the Caco-2 cell layer on the apical side of the insert and the integrity of the barrier during co-culture with THP-1 cells. In our co-culture system, the TEER of the Caco-2 layer increased gradually with the extension of the culture time after plating, reaching the maximum value at day 21. On the 21st day of culture, the TEER reached approximately 550 Ω/cm^2^. The above results indicated that cells cultured for 21 days had formed a complete monolayer.

### 3.4. Effect of SPE on Cell Junctions in the Co-Culture System

**Figure 6:**
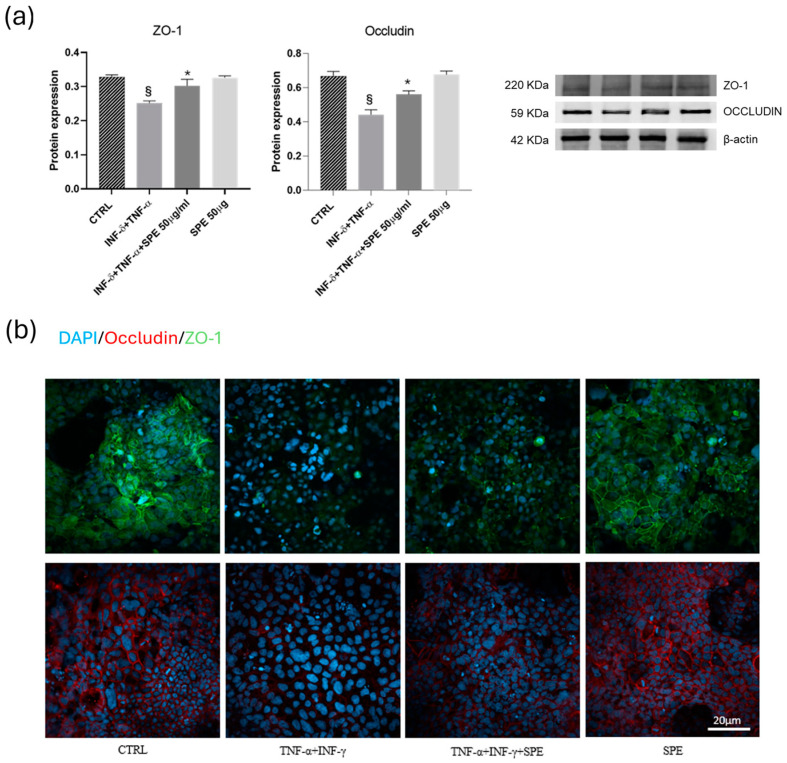
Effects of SPE on the ZO-1 and occludin of Caco-2 in the co-culture system. (**a**) Representative images and quantification of Western blot analysis for the proteins ZO-1 and occludin in each experimental group. Each value represents the mean ± SD (*n* = 3). SPE, Saffron Petal Extract. § *p* < 0.05, vs. control cells and * *p* < 0.05 vs. TNF-α + INF-γ stimulated cells. (**b**) Expression of junctional proteins, occludin and ZO-1, in the apical region of Caco-2 monolayers. Green Colour: immunostaining for ZO-1. Red colour: immunostaining for occludin. Blue colour: colouration of cell nuclei. Bar = 20 µm.

Subsequently, we examine how SPE can help reduce the adverse effects of inflammation on the function of the intestinal barrier and the mechanism behind this. To do this, we measure the protein levels of epithelial TJ proteins. In the Caco-2 cells, the expressions of ZO-1 and occludin significantly decreased after cytokine exposure (Figure 6a). SPE treatment restores these proteins’ expression compared to the activated cells. Consistent with Western blot findings, the intensity of staining for ZO-1 and occludin in TJ increased after petal extract treatment concerning TNF-α + INF-γ activated cells (Figure 6b). These data suggest that SPE preserved barrier integrity by inhibiting the loss of TJ proteins in Caco-2 cells induced by pro-inflammatory cytokines.

### 3.5. Effects of SPE on the Activation of FBW7/NfKB Signaling and Inflammatory Molecules in a Co-Culture System

**Figure 7:**
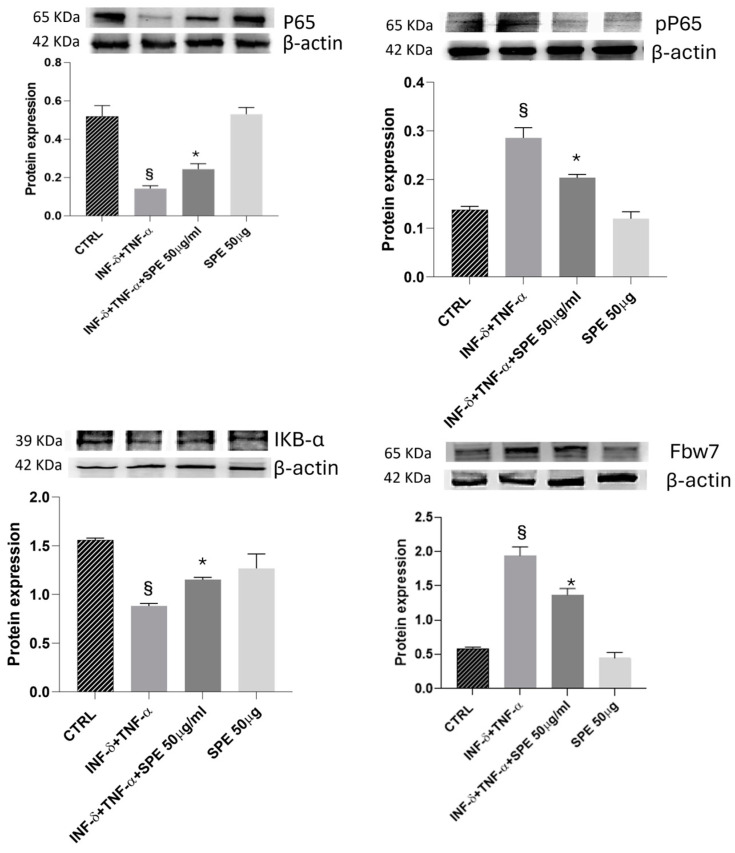
Effect of SPE on Fbw7/NFkB signalling. Western blotting measured the cells’ expression levels of p-p65/p65, IκBα, and Fbw7 proteins. The overexpression of Fbw7 leads to the degradation of IκB α, activating the nuclear factor κB signalling pathway in cytokine-stimulated co-culture. Additionally, treatment with SPE protects cells from proinflammatory stimuli. Each bar represents the mean ± standard deviation of 6 groups of cells. § *p* < 0.05 vs. the ctrl. * *p* < 0.05 vs. INF-γ + TNF-α treated cells. SPE, saffron petal extract; ctrl, control.

Next, we evaluated the impact of SPE on the NF-kB signalling pathway. As shown in Figure 6, P65 expression does not significantly change in the presence of cytokines. However, pP65 was notably upregulated after cytokines stimulus and reversed by SPE pretreatment. As shown in Figure 7, Western blot analysis showed that treatment with SPE inhibited the phosphorylation of p65 and attenuated the nuclear translocation of p65.

Meanwhile, IkBa levels decreased significantly after cytokines treatment and increased with SPE pretreatment, which may result in the enhanced binding of IκB-α to p65. These results suggest that SPE can inactivate the NF-kB signalling pathway, preventing the degradation of IkBa and retaining NF-kB in the cytoplasm (Figure 7). Furthermore, Fbw7 expression was enhanced in the activated co-culture treated with SPE compared to inflamed cells. Fbw7 plays a crucial role in reducing the expression of IKK, inhibiting IkB-α phosphorylation, and suppressing p65 phosphorylation. This concerted action ultimately leads to the inhibition of the NF-kB signalling pathway.

**Figure 8:**
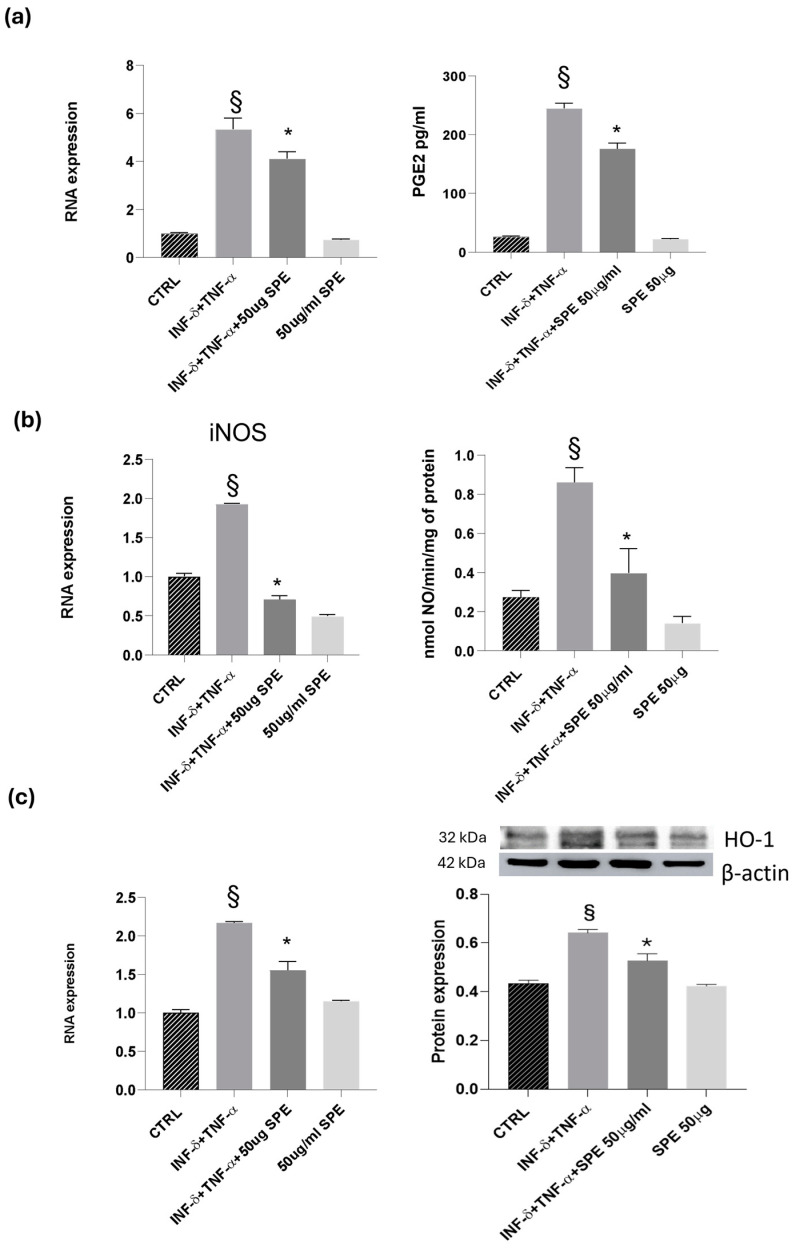
Effect of SPE on COX-2, iNOS and HO-1. (**a**) RNA expression and activity (PGE2 level) for COX-2, GADPH being used as loading control; (**b**) RNA expression and activity (NO level) for iNOS, GADPH being used as loading control; (**c**) RNA expression and Western blot for HO-1, GADPH and actin being used as loading control. Data shown are means ± SD, § *p* < 0.05 compared with control cells; * *p* < 0.05 compared with cytokines treated cells; data were from at least three independent experiments, each performed in triplicate.

Next, we examined the effects of SPE on the inducible molecules as nitric oxide synthase/nitric oxide (iNOS/NO) and cyclooxygenase-2/prostaglandin E2 (COX-2/PGE2). Our results revealed a notable increase in the expression levels of COX-2 and iNOS following cytokine treatment in co-cultured Caco-2 and THP-1 cells (see Figure 8). This upregulation was accompanied by heightened enzyme activity, as reflected in the analysis of NO and PGE2 levels in the culture medium. Cytokine treatment alone led to a noticeable elevation in NO and PGE2 levels compared to the control. However, the addition of 50 µg/mL of SPE significantly reduced the production of NO and PGE2 in cytokine-activated cells when compared to the control group (Figure 8a,b). Notably, in Caco-2 cells co-cultured with THP-1 monocytic cells, SPE exhibited an anti-inflammatory effect by significantly decreasing the levels of the examined molecules. Graphical representations of our findings can be seen in Figure 8. Exposure to high levels of NO activates an additional protein called HO-1, which is crucial for protection. Heat shock protein (HSP) plays a protective role in preventing cell damage from oxidative injury and cytokine-induced cytotoxicity. Activation of HO-1 serves as a natural defence mechanism to decrease inflammation and tissue damage in the gastrointestinal tract. Our study involved examining the mRNA and protein levels of HO-1. Our results showed that reducing NO also decreases the expression of HO-1. We noticed that control cells had a baseline expression of HO-1, which increased in response to cytokine stimulation as a protective measure against cellular inflammation (see Figure 8c). Treatment with SPE restored the baseline expression of HO-1, indicating that the cell no longer needs to protect itself from inflammatory processes.

**Figure 9:**
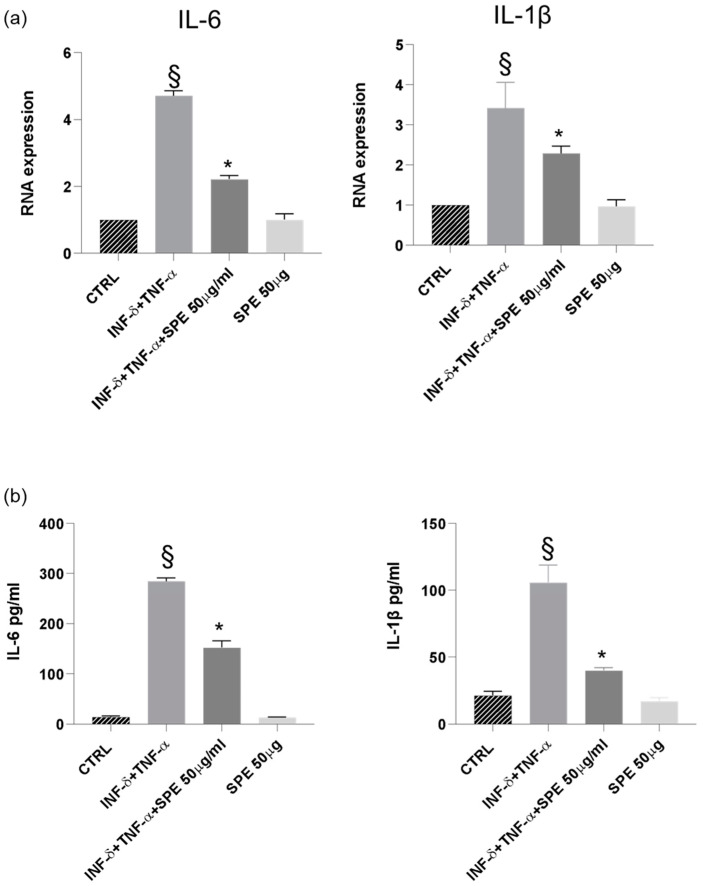
SPE inhibited the production of inflammatory cytokines in THP-1 cells from the co-culture system with Caco-2 cells. (**a**) The expression of IL-1β and IL-6 were analysed using qRT-PCR. (**b**) Commercially available ELISA kits were used to test interleukin (IL)-1β and IL-6 levels in the culture medium. Data shown are means ± SD, § *p* < 0.05 compared with control cells; * *p* < 0.05 compared with cytokines treated cells.

Finally, our results demonstrated a significant downregulation of IL-1β and IL-6 mRNA expression in cells treated with SPE, indicating a suppressive effect on the transcription of these pro-inflammatory cytokines (Figure 9a). Consistent with these findings, a notable reduction in the levels of IL-1β and IL-6 was observed following SPE treatment, suggesting a comprehensive inhibitory effect on both the gene expression and the subsequent production of these key inflammatory mediators (Figure 9b).

## 4. Discussion

*Crocus sativus* is an herbaceous plant used mainly in the food industry to obtain saffron, a widely appreciated spice, from its stigmas. Lately, this noble part of the saffron flower has also been demonstrated to be a source of bioactive molecules such as crocin, crocetin, picrocrocin, and safranal, potentially useful in the health field. Several studies have acknowledged that crocin, extracted from saffron stigmas, has antioxidant and anti-inflammatory therapeutic effects in many diseases [26]. Agri-food industries generate several by-products, including antioxidant-rich materials currently treated as waste [27]. Chronic inflammation and oxidative stress play a vital role in the development and progression of many chronic diseases, such as autoimmune diseases, metabolic and intestinal disorders, cardiovascular diseases, central nervous system disorders, fibrosis, diabetes, obesity, and cancer [28]. In IBD patients, the immune cells release abnormally high levels of pro-inflammatory cytokines compared to healthy patients. The hallmark of Crohn’s disease is chronic inflammation, which is caused and sustained by hyperactivated effector immune cells that increase the production of proinflammatory cytokines, specifically tumour necrosis factor alpha (TNF-α) [29]. These cytokines affect downstream effector cells, including macrophages, that are activated to produce pro-inflammatory mediators such as IL-1β and IL-6. Furthermore, patients with ulcerative colitis and Crohn’s disease exhibit markedly enhanced secretion of these downstream pro-inflammatory cytokines. TNF-α plays a significant role in the pathogenesis of IBD because it can increase the expression of IL-1β and IL-6 [30]. Therefore, blocking the overproduction of cytokines is proposed as an effective treatment for IBD. For mild-to-moderate IBD, the preferred treatments are anti-inflammatory agents, such as aminosalicylates and corticosteroids, and biologic therapies like anti-tumour necrosis factor (TNF)-α agents. The use of anti-TNF-α agents has significantly increased in the last two decades due to their unique ability to alter the natural course of IBD. Both controlled clinical trials and real-world studies have demonstrated that anti-TNF-α agents, including infliximab, adalimumab, golimumab, and certolizumab pegol, can induce and maintain clinical remission while reducing the need for surgery and hospitalisation. Additionally, these therapies have been shown to improve patients’ quality of life and reduce both the direct and indirect costs associated with these chronic, debilitating conditions [31]. However, anti-TNF-α agents are not a universal solution for all cases of IBD. They can cause severe and potentially life-threatening side effects in some patients, primarily due to infectious complications and immunogenicity, which may lead to the formation of antibodies against anti-TNF-α agents and a consequent loss of therapeutic response over time [32]. While aminosalicylates are effective in reducing inflammation, they can also cause side effects, such as headaches, nausea, and diarrhoea, particularly at higher doses. Current treatment strategies mainly focus on managing IBD symptoms but do not address important aspects of the disease, such as promoting mucosal epithelial repair, maintaining barrier homeostasis, or correcting intestinal dysbiosis [33]. IBD pathogenesis is associated with inflammation, epithelial damage, and dysbiosis, leading to a dysregulated gut mucosal barrier. However, the extent and underlying mechanisms remain largely unknown. Intestinal epithelial cells provide a physical and biochemical barrier mediated by TJs. TJs, including occludin and Claudin, are considered the most important mechanical barrier, and their reduction is critical for impaired intestinal barrier function in subjects with chronic intestinal diseases. Several studies have found that inflammatory cytokines lead to damaged tight junctions and a compromised intestinal barrier [34]. Loss of epithelial barrier function and increased epithelial damage lead to mucosal inflammation, so the loss of intestinal TJ proteins is closely associated with the development of IBD. Therefore, achieving mucosal healing is a major treatment goal in managing patients with IBD [35]. There is evidence that oxidative stress and inflammatory processes disrupt epithelial barrier function by downregulating the expression of junction proteins in the gastrointestinal tract [36]. To get a better understanding of how SPE affect the intestinal immune system, we utilised a co-culture model to study the impact on the brush epithelial monolayer in both healthy and inflamed conditions. In this study, we stimulated the Caco-2/THP-1 co-culture models using TNF-α and INF-γ. Cytokines were used as they can reproduce similar cellular responses in IBD, such as increased intestinal tight junction permeability [21,37]. In this study, we show that the SPE bioactive molecules as kaempferol 3-O-sophoroside, quercetin 3-O-sophoroside, isorhamnetin 3-O-glucoside, and kaempferol 3,7,4-O-triglucoside, significantly attenuate epithelial barrier dysfunction, as indicated by the ability of SPE to reduce expression levels of TJ or adherents junction-related genes (ZO-1, occludin) in the co-culture system exposed to proinflammatory cytokines (Figure 6).

NF-kB is an essential and ubiquitous transcription factor for controlling various genes involved in inflammatory responses. Substances with inhibitory activity on the activity of NF-κB p65 effectively attenuated the expression of pro-inflammatory molecules, such as iNOS and COX-2, in macrophages [10,14]. Therefore, the regulatory effects of SPE on NF-κB activation have been studied. In the inactivated cell, the subunit p65 of NfkB is located in the cytoplasm combined with IκB-α, its inhibitor protein. When inflammatory stimuli activate cells, IκB-α is phosphorylated by the IkB kinase (IKK) complex and dissociated from NF-κB, allowing the translocation of pp65 into the nucleus. The translocated subunit promotes subsequent transcription and expression of pro-inflammatory mediators [38]. Our results demonstrated that SPE treatment prevents the interactions of NF-kB p65 with specific genes to mediate an inflammatory response in macrophages. In the co-culture system, treatment with SPE inhibited the phosphorylation of p65, upregulating the protein levels of IκB-α. The enhanced binding of IκB-α with p65 consequently leads to the cytoplasmic retention of the NF-κB complex, which may prevent the accessibility of NF-κB into the nucleus to bind the target gene promoter (Figure 7). The observed inhibitory effect on NF-kB p65 phosphorylation is consistent with the results of Bian et al (2022) in which kaempferol supplementation improved gut barrier integrity and inhibited gut inflammation, reducing activation of the TLR4/NF-κB pathway [39]. Fbw7, a crucial modulator of the NF-kB signalling pathway, has been found to play a protective role in the colitis model. Li et al. demonstrated how Fbw7 inhibits the NF-kB activation induced by DSS treatment. Its inactivation is involved in colitis by modulating the inflammation response of NF-kB signalling in the intestine epithelium. The key findings were the negative correlation between the expression level of Fbw7 and NF-kB signalling activity, and the activation of NF-kB signalling upon Fbw7 deletion [16]. Our experiments further revealed that the downregulation of Fbw7 is related to NF-kB activation (Figure 7). The restoration of Fbw7 expression through SPE treatment underscores its protective role. FBW7, by inhibiting NF-kB activation, is a key protective factor in our experimental model (Figure 7).

Furthermore, the inhibition of NF-kB translocation, induced by SPE, results downstream in a reduced expression of two key inflammatory molecules, iNOS and COX-2, known to be involved in colitis’s pathogenesis [40]. In a co-culture system, treatment with SPE significantly suppressed the secretion of NO and PGE2 by downregulating the expressions of iNOS and COX-2 (Figure 8). This finding is supported by the ability of SPE to inhibit the expression and production of pro-inflammatory cytokines, IL-1β and IL-6, as shown in Figure 9. Our findings support previous studies which investigated the impact of kaempferol and quercetin, molecules present in SPE extract, on colitis in mice. Their research revealed that kaempferol reduced inflammatory markers (IL-6, IL-1β, TNF-α) and enhanced gut barrier function by upregulating ZO-1, occludin, and claudin-1 in mice [41,42,43].

These results suggest that SPE treatment reduces pro-inflammatory mediators by regulation of the NF-κB pathway. In an in vitro co-culture system that mimics the intestinal environment using epithelial Caco-2 cells and THP-1 macrophage cells, SPE treatment exhibited potent anti-inflammatory activity in suppressing cytokines-induced expressions of pro-inflammatory mediators, thereby providing promise for the development of new anti-inflammatory therapies.

## 5. Conclusions

The present study underscores the significant potential of SPE bioactive molecules in treating IBD. The findings demonstrate that these bioactive components can effectively inhibit pro-inflammatory cytokines and mediators by modulating the Fbw7/NF-κB pathway. This inhibition reduces the expression of critical inflammatory proteins, such as iNOS and COX-2, which are implicated in the pathogenesis of IBD. The results of the co-culture model of Caco-2 cells and THP-1 macrophages suggest that SPE treatment, by reducing the destruction of TJs induced by the inflammatory stimulus, restores the functionality of the intestinal barrier. The results obtained from our research present interesting translational perspectives for the use of SPE in the treatment of IBD: (i) SPE could be exploited to develop food supplements or natural product-based drugs aimed at the long-term management of IBD. These could represent a complementary option to conventional drugs, especially for those patients who develop resistance or intolerance to standard therapies; (ii) the use of SPE could be explored in the early stages of IBD or in mild forms, where inflammation and oxidative stress play a predominant role, helping to reduce the need for more aggressive and potentially toxic therapies; (iii) in combination with standard drugs, such as corticosteroids or immunosuppressants, SPE could help to reduce the doses of conventional drugs needed, thus minimizing side effects such as immunosuppression or liver toxicity; and (iv) considering the variability of response to therapies in IBD, SPE could be integrated into a personalised medicine approach. Identifying subgroups of patients particularly susceptible to the anti-inflammatory and antioxidant benefits of this extract could improve the efficacy of treatment. Despite promising preclinical results, several challenges need to be addressed before SPE can be considered an effective therapy for IBD. First, large-scale clinical trials are needed to confirm the efficacy and safety of the extract in humans. Second, the optimal dosage and the most effective formulation to ensure the bioavailability of the active ingredients need to be better defined. Furthermore, possible synergistic or antagonistic effects with currently used pharmacological therapies should be evaluated.

In conclusion, our research provides a solid scientific basis for further studies on the use of SPE as an anti-inflammatory and antioxidant agent in IBD. The translational perspectives are promising and could represent a significant contribution to therapeutic innovation for these debilitating diseases. However, the transition from preclinical research to clinical practice requires further rigorous investigations. Our future research should focus on translating these results to in vivo models to better understand their efficacy and safety in a clinical context.

## Figures and Tables

**Table 1:** Sequence of primers used for RT-qPCR studies.

Gene	Forward Sequence (5′-3′)	Reverse Sequence (5′-3′)	Accession
*FBW7*	CAGTCCGCTGTGTTCAATATG	GCCCTGTTAACGTGTGAATG	*NM_001257069*
*IL-1β*	TGAGGATGACTTGTTCTTTGAAG	GTGGTGGTCGGAGATTCG	*NM_000576.2*
*IL-6*	GAGCTGTGCAGATGATGAGTACAA	GGACTGCAGGAACTCCTTAAA	*NM_000600.3*
*COX-2*	CGATGCTGTGGAGCTGTAT	TTGAGGCAGTGTTGATGATTTG	*NM_000963.2*
*iNOS*	CATTGCTGTGCTCCATAGTTTC	CAGGACGTAAGTTCAGCATCTC	*NC_000017.11*
*HO-1*	TCCACCGGACAAAGTTCAT	CATTGCTGTGCTCCATAGTTTC	*NC_000022.11*
*18s*	CTTTGCCATCACTGCCATTAAG	TCCATCCTTTACATCCTTCTGTC	*NR_003286.2*

## Data Availability

The original contributions presented in the study are included in the article, further inquiries can be directed to the corresponding authors.
